# Reducing psychological distress and obesity in Australian farmers by promoting physical activity

**DOI:** 10.1186/1471-2458-11-362

**Published:** 2011-05-23

**Authors:** Susan Brumby, Ananda Chandrasekara, Scott McCoombe, Susan Torres, Peter Kremer, Paul Lewandowski

**Affiliations:** 1 National Centre for Farmer Health, Western District Health Service, Hamilton Vic 3300, Australia; 2 School of Medicine, Deakin University, Geelong Vic 3217, Australia; 3 School of Exercise and Nutrition Sciences, Deakin University, Burwood Vic 3125, Australia; 4 School of Psychology, Deakin University, Geelong Vic 3217, Australia

## Abstract

**Background:**

Studies have confirmed that the rate of mental illness is no higher in rural Australians than that of urban Australians. However, the rate of poor mental health outcomes, and in particular suicide, is significantly raised in rural populations. This is thought to be due to lack of early diagnosis, health service access, the distance-decay effect, poor physical health determinants and access to firearms. Research conducted by the National Centre for Farmer Health between 2004 and 2009 reveals that there is a correlation between obesity and psychological distress among the farming community where suicide rates are recognised as high. Chronic stress overstimulates the regulation of the hypothalamic-pituitary-adrenal (HPA) axis that is associated with abdominal obesity. Increasing physical activity may block negative thoughts, increase social contact, positively influence brain chemistry and improve both physical and mental health. This paper describes the design of the Farming Fit study that aims to identify the effect of physical activity on psychological distress, obesity and health behaviours such as diet patterns and smoking in farm men and women.

**Methods/Design:**

For this quasi-experimental (convenience sample) control-intervention study, overweight (Body Mass Index ≥25 kg/m^2^) farm men and women will be recruited from Sustainable Farm Families™ (SFF) programs held across Victoria, Australia. Baseline demographic data, health data, depression anxiety stress scale (DASS) scores, dietary information, physical activity data, anthropometric data, blood pressure and biochemical analysis of plasma and salivary cortisol levels will be collected. The intervention group will receive an exercise program and regular phone coaching in order to increase their physical activity. Analysis will evaluate the impact of the intervention by longitudinal data (baseline and post intervention) comparison of intervention and control groups.

**Discussion:**

This study is designed to examine the effect of physical activity on psychological health and other co-morbidities such as obesity, impaired glucose tolerance, hypertension and dyslipidaemia within a high-risk cohort. The outcomes of this research will be relevant to further research and service delivery programs, in particular those tailored to rural communities.

**Trial registration:**

ACTRN12610000827033

## Background

Mental health disorders, particularly anxiety and depression, are the leading cause of health related disability in Australia [1,2]. These disorders make a large contribution to the global burden of disease, with depressive disorders being the leading cause in middle- and high-income regions such as European Union, North America, Japan and Australia [3]. By 2020, depression is projected to reach second place in the global burden of disease rankings [4,5]. Rural Australians face a high mental health burden [6] due to social isolation [7], socio-economic constraints [8,9], poor diet, increased alcohol intake, sub-optimal sleep, lack of exercise, high rates of obesity [10] and diabetes [11]. As a sub-population of rural Australia it has become evident that farmers experience inferior physical and mental health than their rural counterparts [6,12,13]. This is also due to the distance decay effect where the further people are from a service, the longer they wait to access that service [14]. The differences between rural and farmer mental health is highlighted by the increased incidence of suicide in farming communities worldwide [15-17]. A study of South Australian farmers revealed that the suicide rate on farms is 67% higher than in rural populations which, in turn, is higher than in urban South Australia [12]. Ongoing research with the Sustainable Farm Families™ (SFF) program reveals that poor health indicators are pronounced within the farming community [13,17-19]. Furthermore, our previous study [20] has revealed that, farmers had a higher rate of obesity/overweight (64.3%) when compared to national averages. Additionally, the prevalence of abdominal obesity was 8.7% higher than the Australian national average [21]. This study also revealed that 45.9% of the participants were psychologically distressed using the Kessler 10 score [22] of greater than 15, a rate significantly higher than rural Victorians (31.3%), all Victorians (34%) and national (32.7%) averages [6]. Finally, higher levels of psychological distress were positively correlated with abdominal adiposity.

Abdominal obesity has been suggested to be associated with overstimulation of the hypothalamic-pituitary-adrenal (HPA) axis [23,24] due to chronic stress [25], altering diurnal cortisol secretion. Abnormal regulation of the HPA axis and perceived stress-dependent cortisol levels are strongly related to perturbations of the endocrine axis as well as abdominal obesity with metabolic abnormalities [26].

A complex set of interrelationships occur between lifestyle, anthropometric, psychological and physical activity variables [27]. Of particular interest is the apparent relationship between physical and mental health [28,29]. Depressed people generally have lower physical activity levels than people without depression [30,31]. Lower physical activity levels also exacerbate depression resulting in a negative feedback cycle [32]. Conversely, increasing physical activity has the dual benefit of increasing physical fitness and alleviating depression and anxiety [29]. Even without the physical health benefits, increasing physical activity may block negative thoughts, distract people from worries, increase social contact and change the brain chemistry to improve mood [33]. Farming people are seldom thought of as being physically inactive, however the very nature of farming is seasonal, increasingly mechanised, with longer periods of being sedentary and greater reliance on vehicles. Further, opportunities for leisure time activity are reduced since many local sporting clubs have amalgamated or ceased to exist and access to a gymnasium or personal trainers is likely to be difficult due to distance and financial constraints.

The aim of this paper is to describe the pilot testing of the Farming Fit study which will examine the effect of physical activity on psychological health, obesity, impaired glucose tolerance, hypertension and dyslipidaemia in farm men and women.

## Methods/Design

### Intervention community

Inclusion - Farm men and women participating in SFF programs will be recruited. Participants will be recruited from rural Victoria, have been farming for more than 5 years, be aged between 18 and 75 years, speak English and live 10 kilometres or more from a regional centre with a population greater than 10, 000. All study participants will be either overweight or obese as determined by body mass index (BMI) ≥ 25 kg/m^2^.

Exclusion - Participants will be excluded from the study if they have a chronic terminal illness or are pregnant or lactating mothers. People with very high levels of psychological distress will be referred for mental health assessment by a health professional (DASS 21 Score depression > 28 or anxiety > 20 or stress > 37) [33]. Subjects unable to participate in physical activity will not be eligible to participate.

### Preparation for evaluation

**Figure 1:**
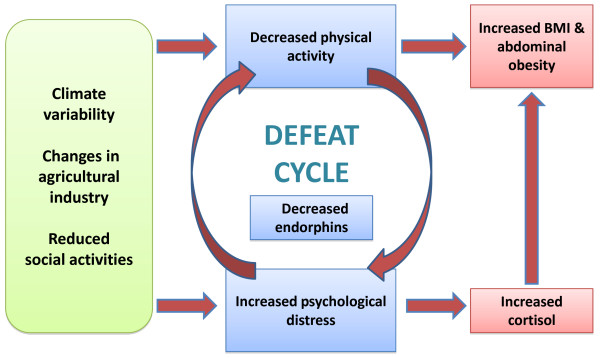
**Farming fit study model illustrating the relationship between psychological distress, physical activity and obesity **[20].

The project design is based on the analysis of data collected by the ongoing SFF program [13]. A logic model of the "farming fit cycle" describes the complex relationship of psychological distress and obesity among farm men and women - see Figure 1[20]. The study protocol detailed here has been specifically designed to measure how increasing physical activity effects pre-intervention variables (anthropometry, biochemistry, nutritional behavior, physical activity behavior and DASS) [34]. Three data collection time points will be utilised to track participant progress (baseline, 12 weeks and 24 weeks).

### Study design

This is a 6-month quasi-experimental control intervention study. Intervention and control group participants whom fulfil the abovementioned criteria will be selected from SFF programs. SFF program groups (clusters) will be randomly assigned to control and intervention groups with participants from each cluster being assigned to the same group to minimise the contamination effect.

The proposed intervention includes:

**1**. An individualised exercise coaching program for the intervention group designed by an exercise physiologist and undertaken over the entire 6-month period.

**2**. An exercise physiologist/research assistant to provide consultation on individualized exercise programs and coach over the 6-month period.

**3**. Regular monitoring of exercise activity and exercise goals by phone, email and/or mobile text message.

Participant's anthropometric and basic physical assessments and psychological assessments (DASS 21 scores) will be measured. Venous blood samples following a 10-hour fast will be taken for biochemical analysis of lipids, glucose and cortisol levels. Salivary cortisol measurements will be taken. Diet and physical activity data will be obtained using self reported questionnaires based on previously developed instruments [35,36]. Data collection will be repeated following completion of the 6-month intervention.

Diet and physical activity questionnaires and salivary cortisol samples are to be repeated following an interim 3-month period of the proposed study as outlined in Table 1. Participants from the intervention group will receive a personalised exercise program and regular phone coaching in order to promote increased physical activity.

**Table 1:** Data collection and timeline for both intervention and control groups

Variable	Base line	3 month	6 month
Body weight (kg)	✓	**X**	✓
Height (cm)	✓	**X**	✓
Waist circumference (cm)	✓	**X**	✓
Hip circumference (cm)	✓	**X**	✓
Body fat (%)	✓	**X**	✓
Blood pressure (mmHg)	✓	**X**	✓
Pulse rate (bpm)	✓	**X**	✓
Fasting blood glucose (mmol/L)	✓	**X**	✓
Fasting blood cholesterol (mmol/L)	✓	**X**	✓
Fasting triglycerides (mmol/L)	✓	**X**	✓
HDL (mmol/L)	✓	**X**	✓
LDL (mmol/L)	✓	**X**	✓
Fasting blood cortisol (nmol/L)	✓	**X**	✓
Salivary cortisol*(nmol/L)	✓	✓	✓
DASS score	✓	✓	✓
Dietary questionnaire	✓	✓	✓
Physical activity questionnaire	✓	✓	✓

* Salivary cortisol will be measured by taking 4 readings/day (9 am, 12 pm, 4 pm and 8 pm)

### Sample size

To evaluate the intervention, sample size was determined at the power of 80% (β = 0.2) with α set at 0.05 for an effect size of a standard deviation change of fasting serum cortisol and/or a 0.5 standard deviation reduction of body weight by the end of the intervention. This required the sample size to be a minimum of 64 participants across the control and intervention groups [37]. By considering the probable retention rate (≥95%) for a 6-month intervention the target recruitment number was fixed at a minimum of 35 participants in both the control and intervention groups, requiring recruitment of a total of 70 farming participants meeting the above criteria.

### Impact and outcome evaluation

Impact and outcome evaluation will be achieved by assessing the immediate and longer term effects of the program. Differences in individual and group impacts and outcomes will be measured using longitudinal (3- and 6-month) data with adjustment for baseline data to evaluate any significant changes across the period of the study. The impact outcome of the anthropometric, biochemical and psychological variables of the intervention will be measured using the quasi-experimental study. Differences between intervention and control groups will be statistically ascertained.

### Data collection

Trained research staff will collect the anthropometric, behavioral, dietary and activity data. They will also conduct physical, behavioral and psychological assessments.

#### 1. Physical Assessment

A complete health history will be obtained from participants registered in the Farming Fit study. Assessments will include anthropometric measures of height, weight, hip and waist circumference, body fat analysis and biochemical analysis of fasted total serum cholesterol, triglycerides, low-density cholesterol (LDL), high-density cholesterol (HDL), blood glucose and cortisol. Salivary cortisol at different time points across a 24-hour period will also be collected.

#### 2. Behavioral assessment

Alcohol habits, activity levels, dietary habits and smoking will be recorded.

#### 3. Psychosocial assessment

A validated questionnaire, the 21-question depression, anxiety, stress score (DASS 21) will be used. The DASS 21 is made up of three seven item subscales covering depression, anxiety and stress as specified in Lovibonds and Lovibond [38].

Socio-demographic, health conditions and medication data will be collected from the participant including age, gender, country of origin, health care card using the Victorian Department of Health Service Coordination Tool Templates (SCTT) [39]. The type of farming undertaken and residential postcode will also be collected from participants.

### Anthropometry

The simplicity of anthropometry allows it to be used in population-based studies to assess body-composition changes over time, as well as in clinical and field based settings where access to technology is limited. Additionally, if used in combination with biochemistry and body fat %, anthropometry can identify distinct fat distribution changes [40,41].

Participant weight will be measured (fasted) to the nearest 0.05 kg using clinically validated A&D™ precision weight scales (UC-321) taken in light clothing with shoes removed and pockets emptied. Height will be measured using a portable stadiometer (S+M™) to the nearest 0.1 cm with shoes removed and weight distributed evenly on both feet. Waist circumference will be measured to the nearest 0.1 cm at the umbilicus at the end of a normal expiration and hip circumference taken at the point yielding the maximum circumference over the buttocks using a constant tension Figure Finder Tape Measure™ [42]. Body Mass Index will be calculated as weight (kilograms) divided by height squared (in meters) to classify obesity and overweight cut-off points [43].

### Physical assessments and health condition data

Physical assessments will be conducted by a trained health professional and will include blood pressure measurement (seated BP measured after at least 5 minutes of rest taken in duplicate one minute apart), pulse rate, body fat percentage (measured by Omron™ HBF 306 Body Logic Pro Body Fat Analyser). Physical assessments will also include a general medical and behavioural assessment which will collect information on alcohol and tobacco consumption.

### Biochemical analysis

#### Lipid profile/fasting blood glucose

Total serum cholesterol, triglycerides, LDL, HDL and fasting blood glucose will be measured to assess metabolic changes associated with abdominal adiposity. Venous blood samples will be obtained following a 10-hour overnight fast. Blood samples will be transported in cold storage and analysed at a pathology biochemical laboratory.

#### Cortisol measurements

The cortisol response is consistently enhanced under chronic stress conditions and studies have demonstrated that the free cortisol response can serve as a useful index of hypothalamo-pituitary-adrenal axis (HPA) activity [26]. The secretion of cortisol, which is a marker of HPA axis activity, follows a diurnal pattern with peak levels following awakening and declining levels thereafter [44]. In this study, fasting blood cortisol will be measured in addition to salivary cortisol. Assessment of cortisol in saliva is a widely accepted and frequently employed method and is considered a valid and reliable reflection of the respective unbound hormone in blood [45]. Due to several advantages over blood cortisol analyses (e.g. stress-free sampling, less intrusive, lower costs), salivary cortisol assessment is the preferred method in basic research and clinical environments. Salivary cortisol is now an accepted, convenient method that is used to provide an indication of the variation of cortisol secretion throughout the day [45]. Salivary cortisol correlates with serum/plasma cortisol concentrations [45] and is stable at room temperature for up to 7-days [46]. Pregnancy, physical and emotional stress, strenuous activity, infection, injury and illness can increase cortisol levels. A number of drugs can also increase levels, particularly oral contraceptives (birth control pills), hydrocortisone (the synthetic form of cortisol), prednisolone and spironolactone. Therefore, the medication history of study participants will also be collected during the study.

Three groups of saliva samples will be collected using the Salivette sampling device (Sarstedt, Rommelsdorf, Germany) at baseline, three months and six months. Participants will be requested to abstain from smoking, ingesting caffeine, alcohol, food and all fluids, and strenuous physical activity for 1 h before each saliva collection. A saliva sample will be collected at approximately 09:00 hour, 12:00 hour, 16:00 hour and 20:00 hour. All saliva samples will be stored at room temperature and then spun at 3,000 × *g *for 5 min within 5 days of collection. Spun samples will be stored at -80°C until saliva cortisol levels are assayed by Radioimmunology assay (Orion Spectra Cortisol^®^).

### Survey methodology

#### Dietary assessment

Dietary intake will be measured using a one-day food record with household measures collected during a common weekday. The food record will be adapted from survey tools developed by the Dieticians Association of Australia [47]. Portion size of commonly consumed foods will be determined by using the Cancer Council of Victoria Food Frequency Questionnaire [48,49]. Daily energy, macronutrient and micronutrient intake will be determined using Foodworks Professional Edition (version 6.0.2539, Xyris Software, Brisbane, Queensland, Australia). Additionally, an 11 item food frequency questionnaire will be used to determine daily intake of fruit, vegetables, soft drinks/energy drinks, water, sweetened beverages (cordial, fruit juice, fruit drinks, and sports drinks) and weekly intake of snack foods. Frequency of daily food consumption will be categorised as follows: no consumption, 1, 2, 3, 4 and 5 serves or more. Frequency of weekly food consumption will be categorised as follows: 1, 2, 3, 4, 5, 6, 7, 8, 9 and 10 serves or more.

#### Physical activity behaviours

Physical activity habits will be measured to assess the influence of this behaviour on body-composition changes. The pre-exercise screening system 2005 of Sports Medicine Australia will be employed to assess the participant's suitability to the particular level of physical activity before personalisation of the program [50]. A questionnaire to collect physical activity data will be prepared by adapting the International Physical Activity Questionnaire (IPAQ) [35,51] and will be administered by a trained researcher at baseline, 3 months and 6 months [35].

#### Depression, Anxiety and Stress Scale - 21 items (DASS 21)

The Depression, Anxiety and Stress Scale - 21 items (DASS 21) is a set of three self-reported scales designed to measure psychological health status [52]. Each of the three DASS 21 scales contains 7 items, divided into subscales with similar content. The Depression scale consists of items that assess dysphoria, hopelessness, devaluation of life, self-deprecation, lack of interest/involvement, anhedonia, and inertia. The Anxiety scale assesses autonomic arousal, skeletal muscle effects, situational anxiety and subjective experience of anxious affect. The Stress scale is sensitive to levels of chronic non-specific arousal. It assesses difficulty relaxing, nervous arousal, and being easily upset/agitated, irritable/over-reactive and impatient. Scores for Depression, Anxiety and Stress are calculated by summing the scores for the relevant items [34,38,52].

The DASS is a quantitative measure of distress, not a categorical measure of clinical diagnoses. However for clinical purposes it can be helpful to have 'labels' to characterise degree of severity relative to the population. Thus the cut-off scores have been developed for defining mild/moderate/severe/extremely severe scores for each DASS scale (Table 2).

**Table 2:** DASS 21 recommended cut-off points [34]

	Depression	Anxiety	Stress
**Normal**	0-9	0-7	**0-14**
**Mild**	10-13	8-9	**15-18**
**Moderate**	14-20	10-14	**19-25**
**Severe**	21-27	15-19	**26-33**
**Extremely Severe**	**28+**	**20+**	**37+**

**Note**: the severity labels are used to describe the full range of scores in the population, so 'mild' for example means that the person is above the population mean but probably still below the typical severity of someone seeking help[34].

#### Data entry, handling and statistical analysis

Data will be tabulated and analysed using PASW^® ^statistics 18 software. Variables will be reported as means with 95% confidence intervals. Repeated measures ANOVA, paired sample t-tests, multiple linear regression, and binary logistic regression will be used to test for intervention effects after adjusting for relevant co-variables (e.g. sex, age) and baseline scores. Since physical activity scores are likely to reflect a non-normal distribution, comparisons between the sexes at baseline and over time will be performed using the Kruskal-Wallis test. Linear regression analysis will be used to assess the association between body fat percentage, body mass index and cortisol levels. Correlation coefficients between body composition, physical activity, and metabolic variables will be calculated using Pearson's and Spearman's correlation as appropriate. P values will be considered statistically significant at P < 0.05.

### Consent and ethics

All adults participating in the study will be provided with a plain language statement and will provide informed written consent. Ethics approval has been granted for the project by Deakin University Human Research Ethics Committee (2009/215) and the South West Multidisciplinary Ethics Committee (HREC 2009/215 dated 03/02/2010). All researchers involved in data collection have had a Victorian Police check undertaken.

## Discussion

Rural populations face poor outcomes in mental health and associated co-morbidities of obesity, diabetes and cardiovascular disease [6-10]. Farming populations are a subset of this group that face additional challenges due to the tyranny of distance, access to health services, stigma associated with mental health issues and the distance-decay effect[6,12,13]. They currently have a high mental health burden and higher rates of obesity and associated health consequences[20]. This study aims to determine the effect of physical activity with coaching on psychological health and biochemical levels of serum cholesterol, triglycerides, LDL, HDL, fasting blood glucose and cortisol in a population of overweight and obese farm men and women. The outcome results of this study will be used in further research and service delivery programs for farming communities.

## List of abbreviations

**SFF**: Sustainable Farm Families™; **K-10**: Kessler 10; **HPA**: Hypothalamus Pituitary Adrenal; **LDL**: Low Density Lipoproteins; **HDL**: High Density Lipoproteins; **DASS-21**: Depression, Anxiety, Stress Score-21; **WC**: waist circumference; **BMI**: Body Mass Index.

## Competing interests

The authors declare that they have no competing interests.

## Authors' contributions

SB was an initiator of the original Sustainable Farm Families program, contributed to the design of the study, and specific health assessment protocols. AC, SM, ST, PK and PL participated in the design of the study and drafting of the manuscript. All authors read and approved the final manuscript.

## Pre-publication history

The pre-publication history for this paper can be accessed here:

http://www.biomedcentral.com/1471-2458/11/362/prepub
